# Imaging findings in a case of situs inversus totalis

**DOI:** 10.1259/bjrcr.20200202

**Published:** 2021-03-04

**Authors:** Kumar Venu Madhav Ramavathu

**Affiliations:** 1 Southend University Hospital, Mid and South Essex NHS Foundation trust, Southend-on-Sea, UK

## Abstract

Situs inversus is an uncommon condition in which there is transposition of thoracic and abdominal structures. We report a case of situs inversus totalis detected incidentally in a 72-year-old female under investigation for haematuria. The imaging findings and role of imaging in this condition have been described in detail. This article emphasises the need for the clinicians to be aware of situs anomalies before planning surgical or radiological interventions in these patients.

## Introduction

Situs inversus is an uncommon condition in which there is transposition of the thoracic and abdominal structures. When this is associated with dextrocardia, it is termed as situs inversus totalis.^1^ As per the literature, there are three types of situs conditions: (1) situs solitus, in which the organs are placed in the normal anatomical position, (2) situs inversus in which the position of the organs is reversed and appear like a mirror image to normal anatomical position and (3) situs ambiguous, which refers to malpositioning of the viscera and dysmorphism in conjunction with indeterminate atrial positioning.^2^ The term situs inversus has originated from a Latin phrase “situs inversum viscerum’’ which means inverted position of the visceral structures. Situs inversus totalis is also referred to as situs inversus with dextrocardia. The heart is present in the right hemithorax instead of left along with a right bilobed and left trilobed lung. The stomach, single spleen and aorta are seen on the right, liver and gallbladder are seen on the left side of the abdomen, instead of right. This condition is present in about 0.01% of the population and is associated with congenital heart disease in 3–5% of the cases. It can occur as a single entity or may be associated with conditions like primary ciliary dyskinesia or Kartageners syndrome (triad of situs inversus, nasal polyposis and bronchiectasis).^2,3^

## Case report and imaging findings

**Figure 1.**
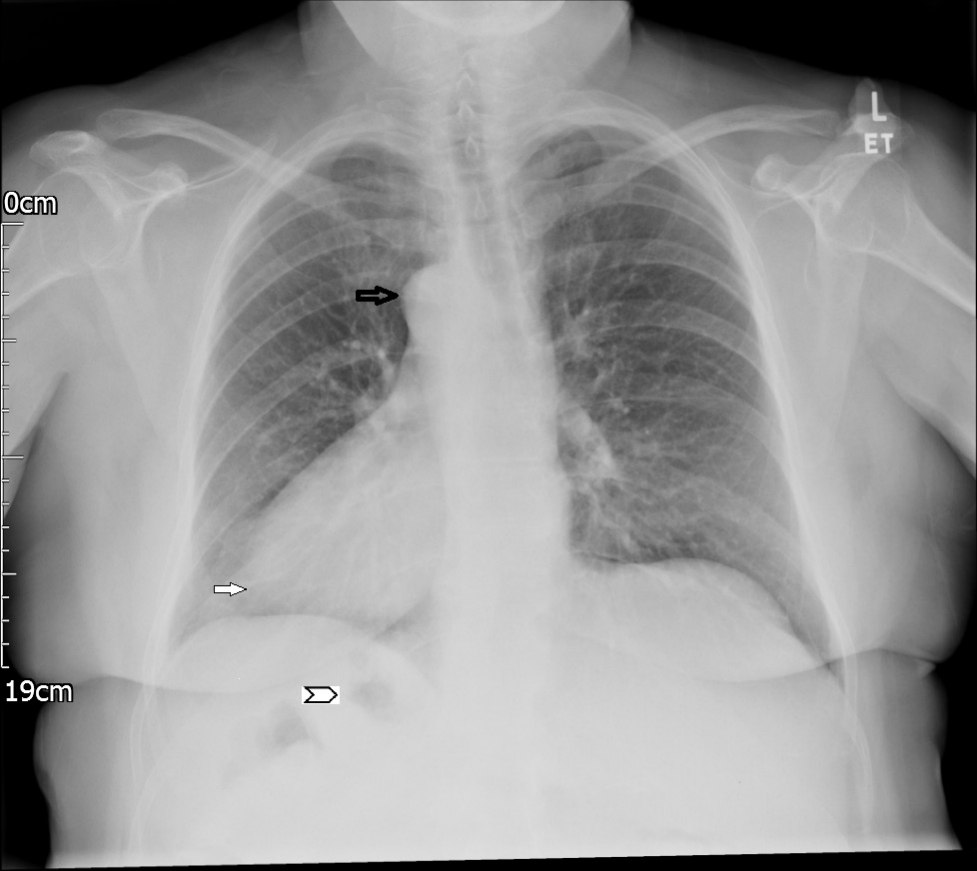
Frontal chest radiograph showing the aortic knuckle (black arrow), apex of the heart (solid white arrow) and gastric bubble (arrowhead) all on the right.

The case that presented to our hospital was a 72-year lady with visible haematuria, who underwent an ultrasound which detected a bladder tumour and subsequently underwent a staging CT chest and urogram. The imaging findings are as follows:

Chest radiograph (Figure 1): the heart is seen to the right (dextrocardia) and stomach bubble under the right hemidiaphragm.

**Figure 2.**
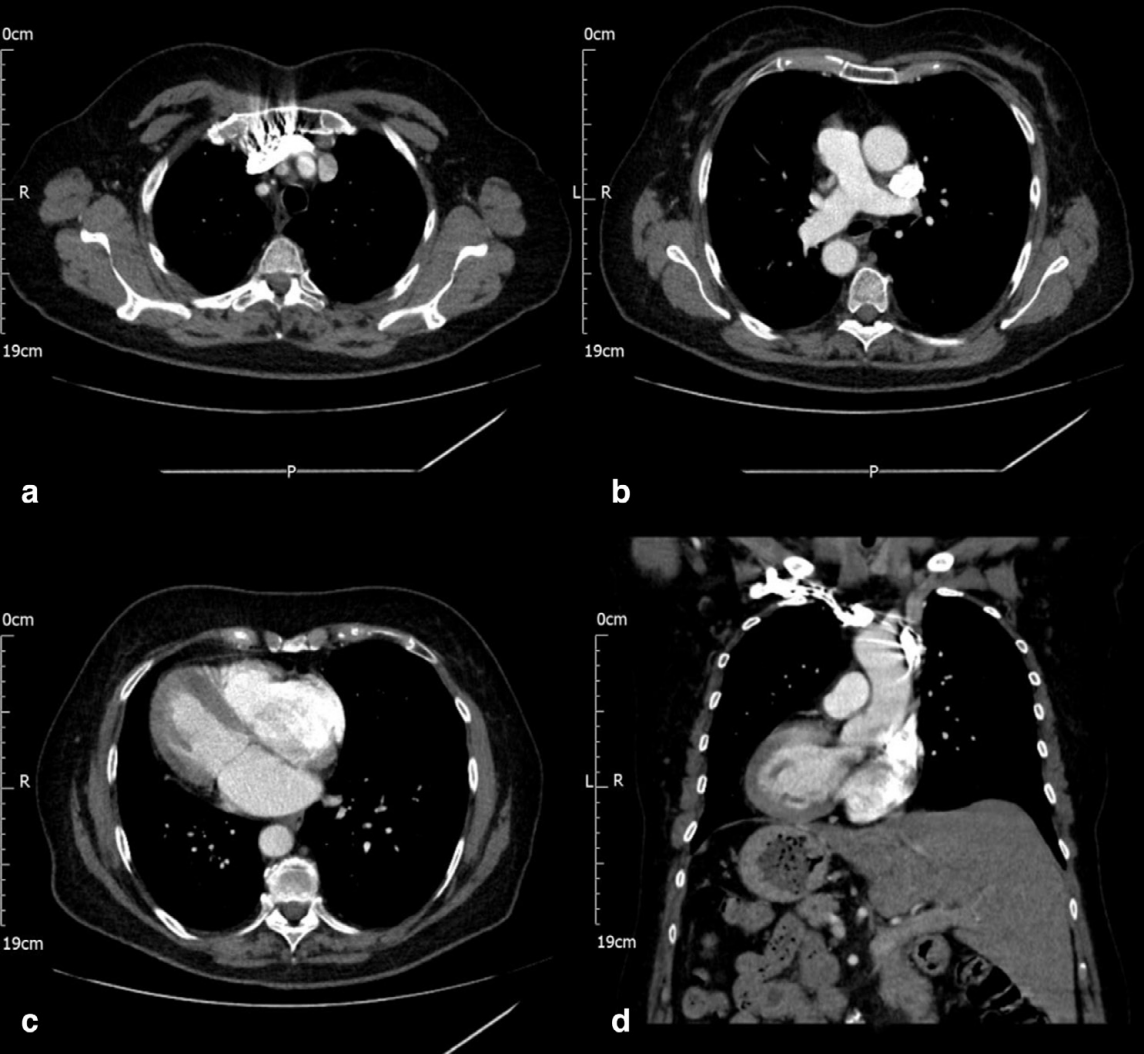
CT scan of the chest shows (a) SVC and brachiocephalic trunk to the left, (b) pulmonary trunk to the right, ascending aorta to the left and descending aorta to the right, (c**, d**) heart in the right hemithorax and liver on the left side of the abdomen. SVC, superior vena cava.

CT scan of the chest:(Figure 2) Trilobed left lung and bilobed right lung, right-sided aortic arch and left-sided pulmonary trunk and heart on the right with morphological right atrium and right ventricle on the left and morphology left atrium and left ventricle on the right. The aortic arch shows two branches on the right, namely the right subclavian and right common carotid and one branch on the left, left brachiocephalic which divides into the left common carotid and left subclavian arteries.

**Figure 3.**
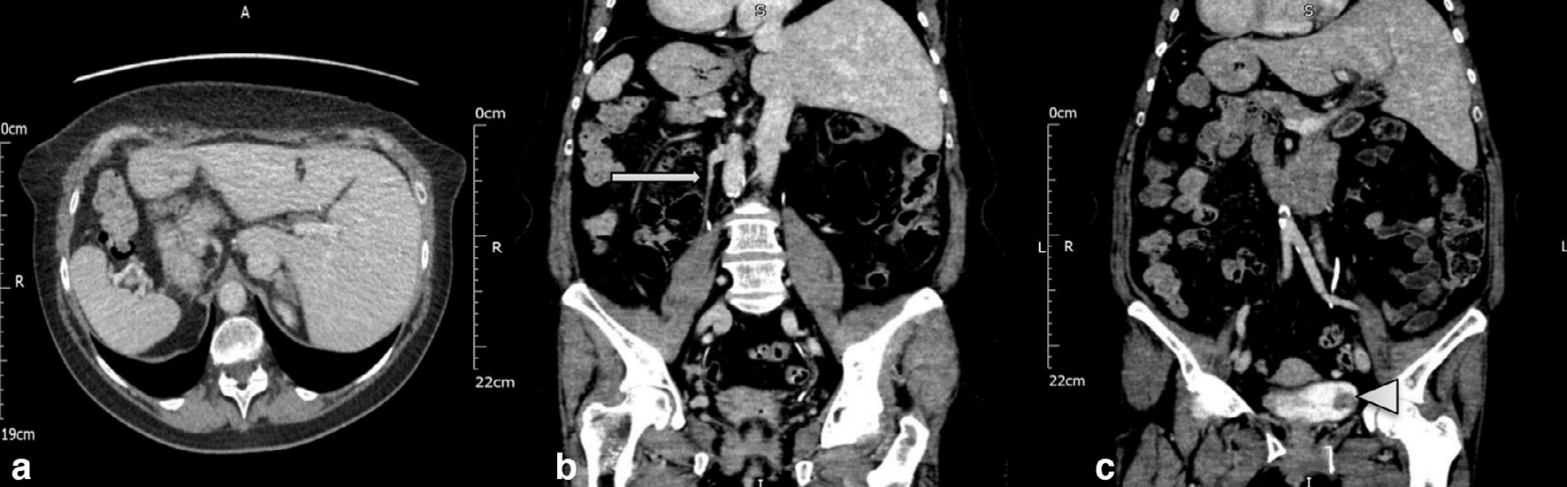
CT in split-bolus excretory phase shows (a) liver, IVC on the left and spleen on the right (b) right gonadal vein opening into the right renal vein (arrow) and (c) tumour in the left lateral wall of the urinary bladder (arrowhead). IVC, inferior vena cava.

CT urogram:(Figure 3) the liver is on the right along with the inferior vena cava; the gall bladder has been resected previously – no previous surgical details available. A single spleen is present on the left. As expected, the bowel shows complete mirror imaging with the stomach, duodenojejunal flexure, descending colon on the right, ileocaecal junction, caecum and ascending colon are present on the left. The right gonadal vein could be seen draining into the right renal vein. Importantly, the patient had a tumour in the left posterolateral wall of the urinary bladder, seen as a filling defect within the contrast filled bladder. She is scheduled to undergo a transurethral resection of the bladder tumour in due course.

## Discussion

Situs inversus totalis is a rare condition and many people are unaware of the condition until they get examined medically for an unrelated condition.^4^ In our case, the patient had a prior cholecystectomy many years ago, but no medical records were available and recently, when she underwent radiological investigations for haematuria, situs inversus was documented. The genetic predisposition of situs inversus is considered to be autosomal recessive with no sex predilection. Although chest radiograph, Barium studies and ultrasound are useful in initially diagnosing this condition, CT and MRI are the modalities of choice for confirmation and outline the full underlying anatomic variations.^5^ On the chest radiograph, the situs is clearly identified by the position of the cardiac shadow and stomach bubble after confirming tthat the marker is not incorrectly placed. , The situs can be confirmed if the bronchial branching pattern is identified on correlating with lateral projection if available.^6^ Cross-sectional imaging like CT with contrast is helpful in identifying the exact anatomical variations in the chest and abdomen, and thus prevents any mishaps at surgical or radiological interventions. CT demonstrates the mirror-image location of the solid organs and a right-sided cardiac apex. Patients with situs inversus also demonstrate mirror image positioning of the bowel and mesenteric vessels in addition to the solid organs. It is important to understand that the orientation of the bowel is reversed rather than malrotated. The positions of the superior mesenteric artery and vein are reversed and the branching pattern of the biliary tract is mirror image to situs solitus.^7^ Situs inversus can occur as an isolated condition or as part of syndrome like Kartagener’s syndrome associated with other abnormalities.^8^ Kartgaener’s syndrome is known to occur as a triad of situs inversus, bronchiectasis and sinusitis and is also known as primary ciliary dyskinesia.^9^ Our patient was not investigated for any genetic disorders as she was asymptomatic with regards to her situs condition. Patients with situs inversus are at risk of diagnostic errors during clinical examination when they present to the Emergency department with conditions like abdominal pain due to unusual sites of pain like left iliac fossa pain for acute appendicitis. Medical imaging by ultrasound, CT and MRI are helpful in establishing the underlying anatomy, particularly before any interventions in order to avoid to potential mishaps.^10^

## Learning points

1. Situs inversus totalis is an uncommon condition often diagnosed lately when patients present to the hospital with unrelated health conditions.
2. It is essential that the clinicians and radiologists are familiar with situs anomalies and a lack of knowledge may pose a considerable danger to the patient if not detected before surgical or radiological interventions.
3. Cross-sectional imaging should be used as a roadmap for establishing the situs anatomy as this information is crucial in planning interventional procedures.
